# The feasibility and impact of instrument-assisted manual therapy (IAMT) for the lower back on the structural and functional properties of the lumbar area in female soccer players: a randomised, placebo-controlled pilot study design

**DOI:** 10.1186/s40814-020-00592-3

**Published:** 2020-04-16

**Authors:** Patrick Weber, Christine Graf, Werner Klingler, Nadine Weber, Robert Schleip

**Affiliations:** 1 Department Movement and Health Promotion, German Sport University Cologne, Cologne, Germany; 2 Anaesthesiology, SRH Hospitals Sigmaringen, Sigmaringen, Germany; 3 Experimental Anaesthesiology, Ulm University, Germany, Ulm, Germany; 4 Clinical Sciences, Queensland University of Technology, Brisbane, Australia; 5 Department Cardiology, Aachen University, Aachen, Germany; 6 Conservative and Rehabilitative Orthopaedics, Department of Sport and Health Sciences, Technical University of Munich, Munich, Germany; 7 Sports Medicine and Health Promotion, Friedrich-Schiller University Jena, Jena, Germany

**Keywords:** Instrument-assisted, Myofascial, Randomised placebo-controlled, Thoracolumbar fascia, Shear motion, Athletes, Ultrasound, Manual treatment

## Abstract

**Background:**

Myofascial (self-)treatments, such as foam rollers to therapeutic instruments in manual therapy, are utilized increasingly in prevention and therapy in healthy people, athletes, and patients suffering from chronic back pain. However, there is limited knowledge about the effectiveness of treatment and the underlying mechanisms of myofascial therapies, especially for instrument-assisted manual therapy (IAMT). Therefore, this pilot study will investigate the feasibility and impact of IAMT for the lumbar area compared with heat application and placebo treatment as a basis for calculating the sample size for further full studies. The primary outcomes will be a critical analysis of the feasibility of the measurement protocol in terms of time economy and expressiveness and of the short- and long-term effects on shear motion of the single tissue layers of the lower back obtained through ultrasound imaging. Secondary outcomes will include thickness and compressibility of the lumbar structures and flexibility of the dorsal structures, indentometry, and superficial skin temperature.

**Methods:**

A minimum of 60 healthy, competitive 15–35-year-old female soccer players will be recruited and randomised into three groups. Short-term effects of IAMT on thoracolumbar structures will be compared with heat application and pressure-less placebo treatment. Long-term effects in the IAMT group will be tested after nine further interventions over a 5-week period (2×/week) and compared with the placebo group, which will not receive further treatments but will serve as a control. Intermediate and final testing of both groups will occur in weeks three and five.

**Discussion:**

This pilot study will assess the feasibility and the impact of IAMT for the lower back particularly by examining the structural and functional properties of myofascial tissue using diagnostic ultrasound. These outcomes could evaluate the feasibility of the measurements used, shall build a basis for sample size calculation of further full studies, and might generate a greater understanding of myofascial therapies, especially IAMT, for the lower back and its benefits. If this approach proves to be practicable, next steps will be further full studies with soccer players, other sports, and patients with low back pain.

**Trial registration:**

German Clinical Trials Register (DRKS00012252) 20.06.2018; retrospectively registered.

## Background

Besides transmitting muscle force, providing structural integrity, and interconnecting the human body and its internal organs [1–4], fasciae have a variety of complex functions. For instance, since the discovery of intrafascial free nerve endings, and Pacini as well as Ruffini corpuscles, fascia is held jointly responsible for nociception and proprioception [5–7]. Further, myofibroblasts and contractile activities have also been observed in fasciae [8]. Because of these various findings, fasciae are increasingly becoming the focus of scientific research in terms of musculoskeletal disorders such as low back pain, which presents one of the most important and cost-intensive complaints [9–12]. In this context, the thoracolumbar fascia plays a central role because it serves as both a pivot and an anchor. It connects the core with the extremities through three different tissue layers [13, 14], and it links the right side with the left side of the body through its crossing fibres [13, 15–17]. Through its connections with the musculi (Mm.) transversus abdominus and obliquus internus, it also acts from the paravertebral muscles down to the deep abdominal muscles [18].

Up to now, most results have come from studies of patients with chronic back pain, in which ultrasound imaging showed structural changes and significant decreases in shear motion of the thoracolumbar fascia [19, 20]. These changes were associated with a negative impact on the mechanical and receptor properties of the fascial tissue, and they may be responsible for limited mobility and flexibility [19–21]. Ultrasound imaging allows an analysis of structural changes like soft tissue consistency and compressibility compared with the established more selective but only mechanical indentometry measurement developed by Fischer [22, 23].

In healthy subjects, Griefahn et al. [24] found a significant increase in shear motion of the thoracolumbar fascia after a single treatment of the Mm. gluteus maximus, latissimus dorsi, and erector spinae with a foam roller in a placebo-controlled trial. Furthermore, the use of foam rollers and massage sticks has been shown to increase the maximum range of motion [25–27], improve vascular functionality [28], and reduce pain without compromising the development of strength, particularly in athletes [25, 26, 29]. In addition to these myofascial (self-)treatments, instrument-assisted manual therapy (IAMT) of the myofascial tissues is being increasingly implemented in usually prescribed regimes of 5 to 10 treatments. For this type of treatment, the therapist applies an appropriate amount of pressure on the myofascial tissue. Specific tools enable the therapist to reach the deeper layers of the tissue and target a more precise or extensive area. This kind of therapy is supposed to increase range of motion and functionality because of improved blood flow accompanied by increased tissue temperature, pain reduction as well as generated pressure, tensile, and shear forces. However, the underlying mechanisms behind these processes are still unknown, but increased microcirculation and altered expression of cytokines in the ground substance, particularly growth factors and inflammatory mediators that enhance regeneration and wound healing, are discussed [21, 30–32].

Through modern diagnostic ultrasound, novel and highly promising findings of both the structural and functional aspects of IAMT are anticipated to provide a greater understanding of its tissue effects.

## Aims

Therefore, this pilot study aims to investigate the feasibility and the impact of IAMT for the lumbar area on the structural and functional properties of the myofascial tissue of the lower back and adjacent motion segments in healthy female soccer players as a reference for sample size calculation of further full studies.

The primary outcomes will be a critical analysis of the time economy and expressiveness of the used measurement methods in terms of quality and quantifiability as well as the effect of IAMT on shear motion of the lumbar structures (subcutaneous tissue, fascia, and musculus (M.) erector spinae) obtained through ultrasound imaging and compared with heat application (short-term) and placebo treatment (short-term and long-term). Secondary outcomes will include thickness and compressibility of the lumbar structures as well as flexibility of the dorsal structures, mechanical tissue deformation resistance (indentometry), and superficial skin temperature. Further secondary outcomes will be the subjectively perceived flexibility and state of the lower back.

## Methods

### Study design

**Fig. 1:**
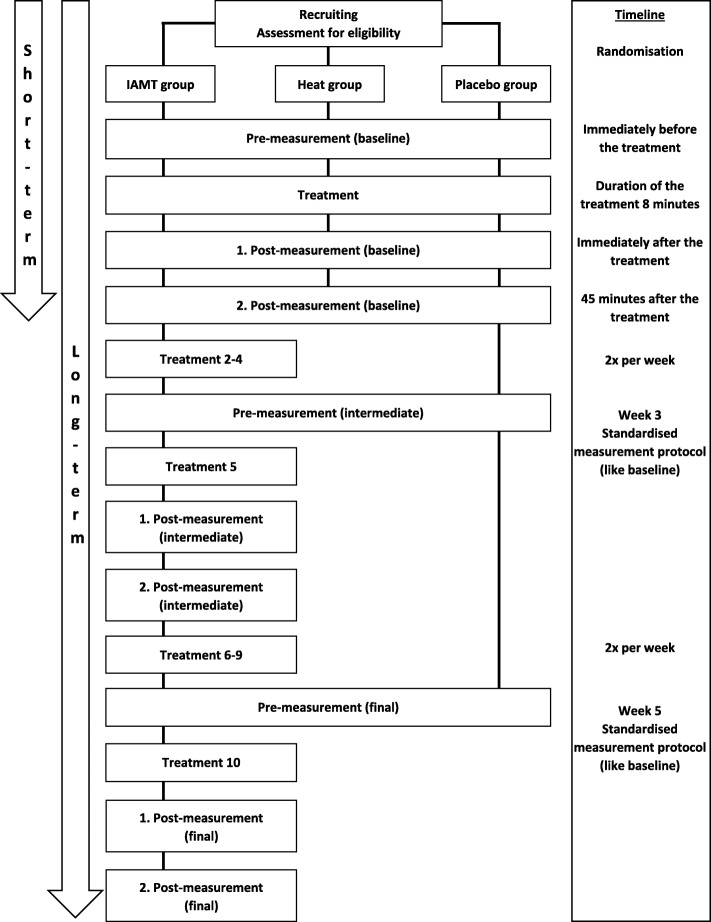
Schematic representation of the pilot study design

**Fig. 2:**
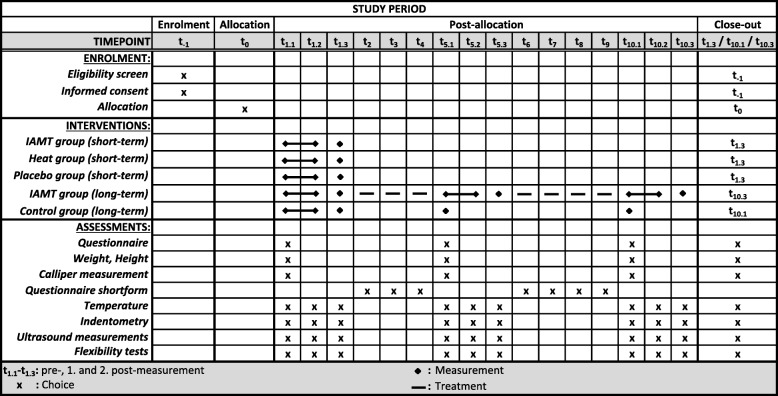
SPIRIT-Figure

This randomised placebo-controlled pilot study will evaluate the feasibility of the planned measurements and generate the first information about the underlying effects of IAMT on structural and functional tissue properties of the lower back as recently reported in a pre-existing study on the lower leg [32]. Subjects will be recruited by a person who is not involved in the testing. The participants will be randomised into three even groups (1:1:1) using an adaptive biased-coin design (urn design), and they will receive an anonymised participant ID. The intervention group (IAMT group) will receive a standardised IAMT on the lumbar area. The comparison group (heat group) will receive heat therapy through an applied heat source, whereas the third group (placebo group) will undergo a pressure-less placebo treatment using an ultrasound transducer. All treatments will be performed once on the right side of the lower back by the same therapist. Testing, using a standardised protocol, will be conducted immediately before, immediately after, and 45 min after the treatment by a blinded investigator who is not involved in the treatments. The long-term evaluation will start with the first treatment, after which the IAMT group will receive nine further IAMT interventions twice a week over a five-week period, as usually practised in Germany, with a minimum of 48 h between treatments. The long-term effects of the IAMT will be controlled by the placebo group, which will be tested regularly over the 5-week period but not treated any more after the initial treatment and will act as a control group for the long-term effects of IAMT from this timepoint. Intermediate and final testing will be performed in weeks three and five, and a short questionnaire about subjectively perceived changes of flexibility and state of the lower back, as well as deviations in frequency and duration of the weekly training schedule, will be distributed before every intervention (Figs. 1 and 2).

### Informed consent, ethics approval, and confidentiality

Prior to commencing this pilot study, subjects will be informed verbally and in writing about the procedure and the aims of the study, and they will provide informed consent. All data collected, including images and videos, will be anonymised and saved on a password-protected USB drive. Individual data will not be accessible by third parties, and they will only be published anonymously. This pilot study has received ethical approval from the German Sport University Cologne (no. 80/2017) in accordance with the latest version of the Declaration of Helsinki.

### Blinding

Participants in the placebo group will assume that they have received appropriate treatments and will be informed about the placebo treatment only after this pilot study is finished. All treatments will be performed by one therapist, while all outcome measures will be assessed by another, blinded investigator. All measurements will be carried out through a standardised protocol, and they will be blind analysed a few weeks after data collection is finished.

### Participants

A minimum of 60 senior-level female soccer players aged 15–35 years, who practise at least three times a week for 90 min and are actively involved in competition, will be recruited for this pilot study. Participants must be healthy, must not suffer from chronic illness or an acute injury of the locomotor system, and must not have had back pain or any other back issue within the past 4 weeks. Because of the study investigator’s work at the elite women soccer team of Cologne, these players present a reachable collective besides their homogeneous and open-minded characteristics to evaluate the feasibility of the planned measurement protocol and generate first results on IAMT. Athletes will also be recruited from the numerous high-level women’s soccer clubs around Cologne and the German Sport University Cologne in order to achieve the desired sample size.

### Measurements

**Fig. 3:**
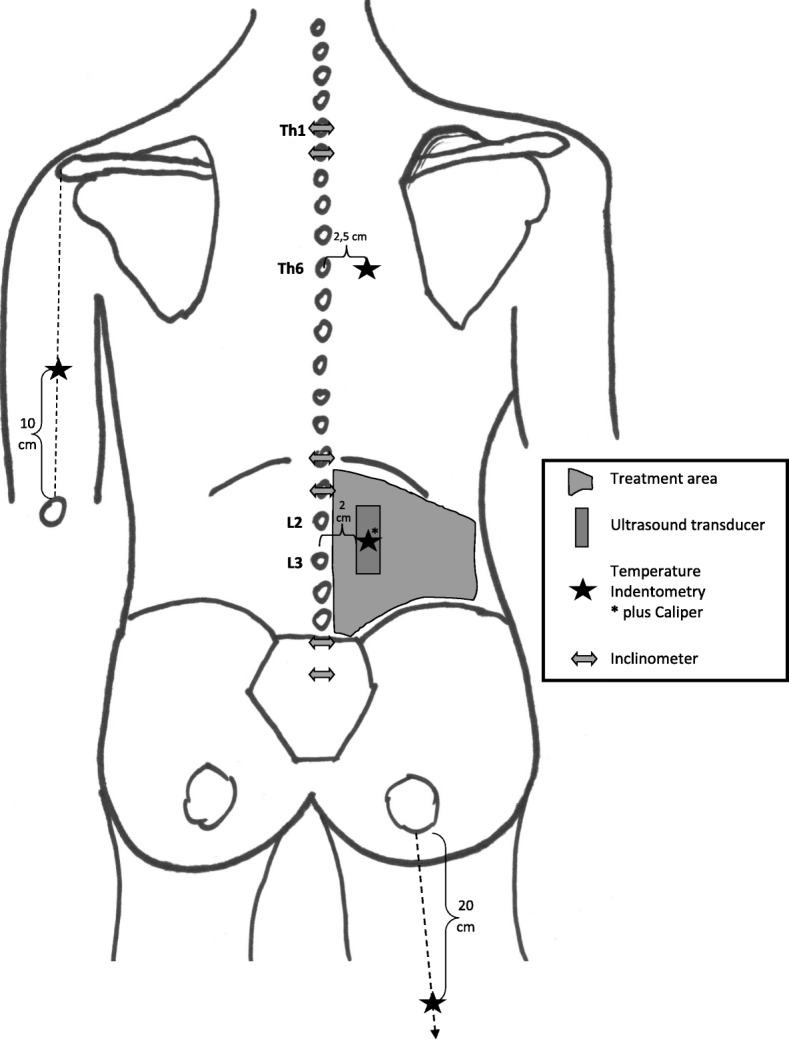
Schematic representation of the measurement points on the treatment side

First, anthropometric data including height and weight will be obtained, and information about the athletic career, individual lifestyle, training sessions, and subjectively perceived flexibility and state of the lower back will be collected through a questionnaire to identify differences between the three groups. Skinfold thickness of the lower back will be measured using a skinfold calliper. After a 2-min rest, both sides of the body will be tested using the following order of measurements: superficial skin temperature, mechanical tissue deformation resistance (indentometry), shear motion, thickness, and compressibility of the single tissue layers, as well as flexibility of the dorsal structures. Every athlete will then receive one of the three different types of treatment and undergo post-measurements. Testing will alternate between body sides, starting with the treatment side (Figs. 1, 2, and 3). All measurements will be taken three times in a row, except for ultrasound imaging and flexibility measurements. Mean values will be used for comparative analysis.

### Preparing and positioning the participants

Participants will undress to their underwear, and the lumbar region and any body part receiving measurements will be cleaned and marked (Fig. 3). All measurements will be conducted with the participant in a prone position on a horizontal treatment table (Stockholm, Clap Tzu GmbH), except for flexibility testing. For all treatments, the treatment table will be pivoted into a 45° roof position to prestretch the myofascial tissue. The anterior superior iliac spine (ASIS) will be positioned above the rotation axis between the lower and mid-section of the therapy table. The participant will lie on the treatment table facing the floor, with arms lying next to the upper body, legs stretched out, and feet supported by a 10-cm-long, half-round foam roller.

### Outcomes

#### Feasibility evaluation

The evaluation of feasibility will investigate the time economy and expressiveness of the measurement methods used in terms of quality and quantifiability. In this context, it will also be determined whether all the measurements are required for further investigations. Therefore, the results of the ultrasound techniques will be compared with the already more established methods to analyse their dependability and accuracy. Shear motion analysis will be compared with the more established flexibility tests and the analysis of compressibility with the mechanical tissue deformation resistance (indentometry). Furthermore, the ultrasound measurements will be assessed by the investigator in terms of their qualitative presentability and their related analysis procedures regarding to their manageability by using a five-level Likert-type scale (from very good to very bad) after each measurement. All measurement methods will be evaluated for their time economy by measuring the time needed for each measurement process. Also, the measuring instruments used will be critically ranked by the therapist and the investigator using a five-level Likert-type scale (from strongly agree to strongly disagree). The ranking will be based on the recorded outcomes to evaluate whether they are suitable to assess short-term and long-term changes in the selected treatments and at the chosen measurement time points. Afterwards, both experts will be asked for suggestions to optimise and adapt the study protocol.

#### Ultrasound scan

For the ultrasound measurements (Sonido Smart Plus, Zimmer MedizinSysteme), the medial aspect of a linear 3.8-cm-long transducer will be placed longitudinally 2 cm lateral to the lumbar spinous processes, midway between lumbar vertebrae (L) 2 and L3 (Fig. 3). Measurements will be taken using a cine-loop technique with a single ultrasound beam focused on the thoracolumbar fascia with a 3.2 cm depth. During the measurement, the therapy table will pivot into the 45° position at a speed of 3°/s. The thickness of the single tissue layers in the starting and end position, as well as the shear motion between these positions, will be measured in millimetres (mm) [19, 20, 24]. The compressibility of the lumbar structures will be measured in the starting position using a force gauge attached to the transducer. An ultrasound image will be taken while applying 0, 5, and 10 Newtons (N) (± 0.25) of force onto the transducer and a measurement of the thickness of each single tissue layer will be taken in millimetres [22].

#### Flexibility tests of dorsal structures

Hamstring flexibility will be tested through the passive straight leg raise test. The participant will lie supine while the investigator passively raises the extended leg angular into hip flexion until a noticeable pelvic rotation and/or resistance is observed. Simultaneously, the investigator will palpate the ASIS of the ipsilateral pelvic side and fix an inclinometer (AcuAngle, Baseline) at the lateral malleolus level. Range of motion will be measured in degrees when a maximum position is reached [33, 34].

The flexibility of the lumbar spine will be measured using a double inclinometer method. With the participant standing, one inclinometer will be placed at the base of the sacrum and the other will be placed in the middle of the motion segments at the spinous processes of the thoracic vertebra (Th) 12 and L1 (Fig. 3). From a standing position, the participant will perform maximal spinal flexion, first eccentrically then concentrically, while keeping the legs extended. To do so, the participant will allow the upper body to bend down eccentrically with gravity. After that, she will be asked to engage muscles actively to reach a maximum range of motion. The difference between the sacral and the thoracolumbar inclinometers will determine lumbar flexibility in degrees [20, 35–37]. The flexibility test of the thoracic spine will follow the same protocol. The inclinometer at Th12 and L1 will be kept in position, while the second inclinometer will be placed onto the spinous processes of Th1 and Th2 [38–41].

#### Indentometry

Indentometry (IndentoPRO, Technische Universität Chemnitz) will be used to measure mechanical tissue deformation resistance at four different sites. Measurements will be taken within the treatment area 2.5 cm lateral to the interspinous space of the spinous processes L2 and L3. Further measurements will be taken 2.5 cm lateral to the spinous processes of Th6 and 20 cm distal to the ischial tuberosity on a connection line with the midpoint of the popliteal fossa. A reference point will be determined on the M. triceps brachii of the contralateral side 10 cm cranial to the olecranon on a connection line with the dorsolateral corner of the acromion (Fig. 3). The indentometry pin (1 cm^2^ surface area) will be put onto a measurement point with the circular reference plate (7.5 cm diameter) placed around it onto the surrounding tissue. Force will be applied at a constantly increasing rate of 5 N/s. At depths of 9 mm and 10 mm and at the pressure pain threshold (algometry), the applied force will be measured in N [23, 42–44]. In addition, during the pressure pain threshold, the indentation depth in mm will be registered. Pressure pain threshold is defined as an increasing sensation of pressure and a transition into feeling discomfort causing stinging, aching, drilling, or burning sensations that are clearly different from the feeling of pressure [44].

#### Superficial skin temperature

Superficial skin temperature will be measured in degrees Celsius at the same four sites as previously used for indentometry by using a multifunctional thermometer (FT70, Beurer Medical).

### Treatments

The treatment area stretches from the 12th rib down to the iliac crest and from the spinous processes to a line connecting the costal arch with the iliac crest.

#### IAMT group

**Fig. 4:**
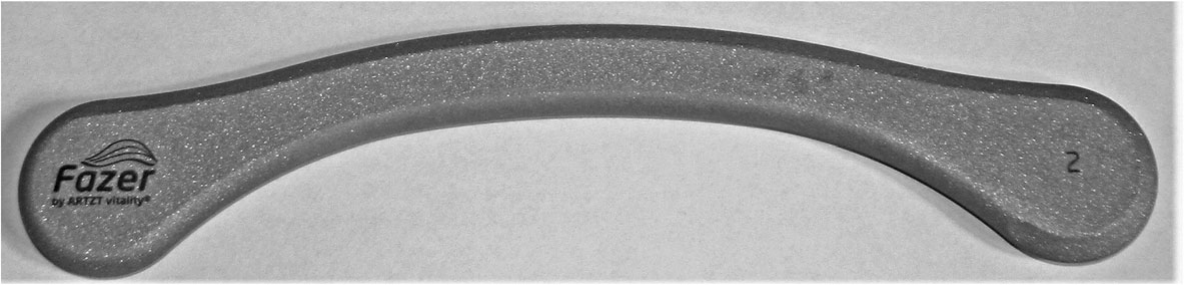
Fazer 2 by Artzt vitality (Ludwig Artzt GmbH)

**Fig. 5:**
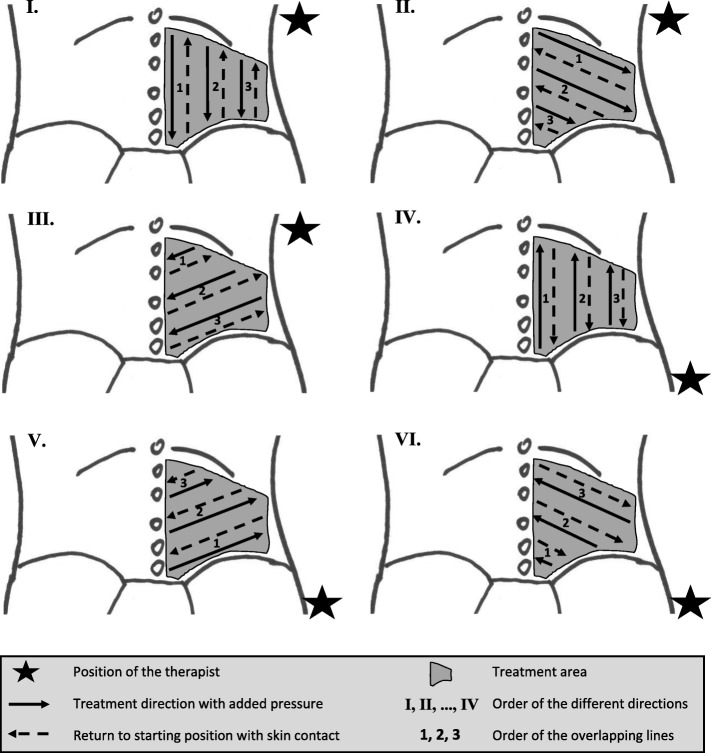
Schematic and chronological order of the instrument-assisted manual therapy techniques

IAMT will be performed using two different techniques and applying Fazer number two (Fig. 4) made by Artzt Vitality (Ludwig Artzt GmbH). The Fazer is made of medical stainless steel and is built to reach deeper structures better as well as treating broader tissue areas. It is a handy and ergonomic tool for the therapist to use, and it allows for more specialised and standardised treatment. The first technique will consist of short, shock-like frictions in the direction of movement. For the second technique, the Fazer will be slowly moved through the tissue creating a skin fold in front of it. Both techniques will be performed in only one direction. The convex side of the Fazer will be applied to reach, but not exceed, the pressure pain threshold. Skin contact will be maintained when returning the Fazer to the starting position. Both techniques will be applied in six different directions: first, from cranial to caudal longitudinally, then diagonally from medial to lateral, and finally from lateral to medial, followed by the same three directions from caudal to cranial. In every direction, the therapist will treat three overlapping lines. The lines will be completed one after another, from the medial to the lateral longitudinal line and starting from the diagonal line closest to the one furthest away from the therapist. After completing all three lines, a second round will be performed before the therapist continues with the next direction and the second technique (Fig. 5). To ensure quality standards, the intervention will be monitored by video. A lubricating film of pH-neutral cream will be applied to the treatment area to reduce frictional resistance and avoid skin redness.

#### Heat group

Heat will be applied through a hot pack with a surface area of 15 cm^2^ and an initial temperature of 55 °C (± 2.5) for a total of 8 min (treatment duration of the IAMT group).

#### Placebo group

Placebo treatment will be performed without pressure over a duration of 8 min using an ultrasound transducer (Sono 5, Zimmer Elektromedizin) at 0.0 W/cm^2^. Using a conductive gel, the therapist will move the transducer (5 cm^2^) over the treatment area in a circular motion with moderate pace and without added pressure.

### Statistical analysis

Statistical analysis will be performed with SPSS Statistics 25 (IBM). Usual descriptive statistics will be used to summarise the results. Means per group, mean differences between the groups will be outlined with standard deviation, 95% confidence interval, minimum, and maximum. Baseline sample characteristics of the groups will be compared by using a single factor analysis of variance or in case of missing assumptions with the Kruskal-Wallis test. Qualitative variables like experiences in myofascial self-treatments and “preferred free leg” will be analysed by using a chi-squared test. Main outcomes like shear motion (distance in mm) and flexibility (range of motion in degree) will be analysed for the three measurement time points in the short-term evaluation as well as for all measurement time points of the IAMT group and the control group in the long-term evaluation by using a longitudinal generalised linear mixed model. Those analyses will include groups, time (measurement time points), and group by time interaction as fixed effects, each participant as random effect, and a covariance structure that provided the best fit for the model to obtain estimates of slopes before and after the treatment and compared between groups like age, experiences in myofascial self-treatments, and “preferred free leg”. The Kruskal-Wallis test will be used to compare the experts ranking of suitability of the measuring instruments, whereas the time economy of the measurement instruments used will be analysed by using a single factor analysis of variance, if the necessary assumptions will be met. To evaluate the quantitative expressiveness of the ultrasound measurements with those of the flexibility tests and the indentometry, their relationship will be analysed by Pearson’s correlation coefficient, if the outcomes will be distributed normally, otherwise Spearman’s correlation coefficient will be applied. The significance level for all tests will be 5%.

## Discussion

To the best of the author’s knowledge, this proposed pilot study will be the first investigation to assess the feasibility and the quantitative impact of IAMT for the lower back on both structural and functional levels. The primary aims will be to evaluate the measurement methods used in terms of time economy and expressiveness and to create a basis to justify the sample size of future full studies.

The comparison of the ultrasound examinations with the previously more established indentometry and flexibility tests should provide information about the qualitative and quantitative properties of the novel ultrasound techniques especially in terms of their dependability and accuracy. Those results, in combination with the time economy and evaluation of the experts, are supposed to verify whether all the used measurements are necessary for future full studies.

To classify and quantify potential therapeutic effects of IAMT, possible confounding factors of the intervention, such as increased local blood flow and resulting tissue reactions or a therapist effect will be controlled by the heat group and by the attention and care the therapist gives to the placebo group. Modern and innovative techniques of ultrasound imaging initial and specific determination of thickness, compressibility, and shear motion of the single tissue layers might make it possible to examine the structural and functional properties of myofascial tissue after IAMT. These novel results may crucially provide a greater understanding of myofascial therapy, especially in IAMT of the lower back, and the potential impact of such therapy on myofascial chains. Moreover, the results may shed light on the mechanisms behind the effects of IAMT on the myofascial tissue and on the functional properties of the human body, for example, receptor stimulation or release of adhesion and fibrosis. This would allow for a better understanding of the benefits of IAMT and its subjectively perceived effects.

In this pilot investigation, modern and objective testing methods will be applied first to healthy female soccer players to evaluate the feasibility of the planned measurement protocol and treatment timeframes in the context of IAMT and, if necessary, to adapt them. Furthermore, the information generated about the effects of IAMT should build first references for the sample size calculation of further full studies. If this study protocol proves feasible to implement, the next step will be a full study. Then, studies of male soccer players and of athletes from other sports would follow. In addition, the effects of IAMT on both athletes and non-athletes with low back pain and its potential impact on strength development in healthy athletes, present an exciting spectrum of future full studies.

## Data Availability

Data sharing is not applicable to this article as no datasets have been generated or analysed to date. As soon as data have been collected and analysed, they will be available from the investigator. Results of this randomised placebo-controlled pilot study shall be published separately, once for the short-term effects and once for the long-term effects, as main articles in an appropriate journal. Results will be made available for interested colleagues through congress presentations. Moreover, every participant will receive a summary of the final study results.

## Abbreviations

ASIS: Anterior superior iliac spine
IAMT: Instrument-assisted manual therapy
L: Lumbar vertebra
M.: Musculus
Mm.: Musculi
Th: Thoracic vertebra
